# Molecular analysis of subtilase cytotoxin genes of food-borne Shiga toxin-producing *Escherichia coli* reveals a new allelic *subAB* variant

**DOI:** 10.1186/1471-2180-13-230

**Published:** 2013-10-15

**Authors:** Joschua Funk, Helen Stoeber, Elisabeth Hauser, Herbert Schmidt

**Affiliations:** 1 Department of Food Microbiology, Institute of Food Science and Biotechnology, University of Hohenheim, Garbenstraße 28, Stuttgart, D-70599, Germany

**Keywords:** Subtilase cytotoxin, Shiga toxin-producing *Escherichia coli*, *subAB*, Deer, New variant, Complete open reading frames, Phylogenetic heterogeneity

## Abstract

**Background:**

The open reading frames of *subAB* genes and their flanking regions of 18 food-borne Shiga toxin-producing *E. coli* (STEC) strains were analyzed.

**Results:**

All but one *subAB* open reading frames (ORF) were complete in all STEC strains. The *subAB*_*1*_ genes of nine STEC strains were located on large plasmids. The *subAB*_*2*_ allele (here designated *subAB*_2-1_), which was recently described by others to be present in the Subtilase-Encoding PAI (SE-PAI) was found in 6 STEC strains. A new chromosomal *subAB*_2_ variant, designated *subAB*_2-2_ was detected in 6 strains and was linked to a chromosomal gene hypothetically encoding an outer membrane efflux protein (OEP). Three STEC strains contained both *subAB*_2_ variants. DNA analysis indicated sequence conservation in the plasmid-located alleles and sequence heterogeneity among the chromosomal *subAB*_*2*_ genes.

**Conclusions:**

The results of this study have shown that 18 *subAB*-PCR positive STEC strains contain complete *subAB* open reading frames. Furthermore, the new allelic variant *subAB*_2-2_ was described, which can occur in addition to *subAB*_2-1_ on a new chromosomal locus.

## Background

Shiga toxin-producing *E. coli* (STEC) can cause serious human infections ranging from uncomplicated watery diarrhea to bloody diarrhea, up to the hemolytic uremic syndrome (HUS), including neurological complications [1]. The production of Shiga toxins (Stx) is considered to be the major virulence factor of STEC [2]. In addition to the production of Stx, the generation of histopathological lesions on host enterocytes, termed attaching and effacing lesions, which are caused by proteins encoded on the locus of enterocyte effacement (LEE) can lead to serious symptoms of disease [3]. The intimin-encoding *E. coli* attaching and effacing (*eae*) gene is located on the LEE. Intimin is involved in the intimate attachment of STEC to the enterocytes, and the corresponding *eae* gene has been used as a marker for the presence of the LEE [4]. In contrast, *eae*-negative *E. coli* of various serotypes were described to cause serious diseases. Examples of these are the outbreak of hemolytic-uremic syndrome (HUS) caused by a STEC strain of serotype O113:H21 in South Australia in 1998 [5], and more recently, the serious outbreak of diarrhea and HUS in Germany in 2011 with STEC of serotype O104:H4 [6]. Such strains may harbor other important virulence markers than the LEE. Whereas the O104:H4 outbreak strain had an enteroaggregative *E. coli* backbone, the O113:H21 outbreak strain expressed a subtilase cytotoxin (SubAB) with cytotoxic and apoptotic properties, in addition to Stx [7]. Paton et al. [8] described this novel AB_5_ cytotoxin occurring in the *eae*-negative STEC O113:H21 outbreak strain. This toxin caused cell death in a number of animal and human cells and enhanced survival of pathogenic *E. coli* strains in macrophages [9]. The initially described subtilase cytotoxin SubAB is encoded by the closely linked *subA* and *subB* genes organized in an operon structure on the megaplasmid pO113 [7,8]. The STEC autoagglutinating adhesion *saa* is also located on pO113, close to the *subAB* operon [8].

This subtilase cytotoxin consists of a single enzymatic active A-subunit (SubA) and five receptor binding B-subunits (SubB). SubA comprises 347 amino acids and contains the catalytic triad Asp-52, His-89, and Ser-272 typical of subtilase family serine proteases [8]. The SubB protein is 141 amino acids in length and responsible for the receptor mediated cellular uptake. SubA is a serine protease cleaving the chaperone GRP78/BiP in the endoplasmatic reticulum (ER) [10]. This leads to an unfolded protein response and ER stress-induced apoptosis [11]. Moreover, it has been demonstrated that SubAB confers HUS-like symptoms in mice [8,12]. SubB has a high binding specificity for α2-3-linked N-glycolylneuraminic acid (Neu5Gc), and a lower binding specificity to α2-3-linked N-acetylneuraminic acid (Neu5Ac) [13]. Human cells are not able to synthesize Neu5Gc but can generate high affinity receptors when incubated with this molecule [14]. It has been hypothesized that ingestion of Neu5Gc rich diet will confer susceptibility to the SubAB toxin [13].

Besides the plasmid-located *subAB* (*subAB*_*1*_) operon, a chromosomal variant was described in 2010 by Tozzoli et al. [15]. This variant (*subAB*_*2*_) showed only 90.0% sequence identity to the plasmid-located one but was also able to cause cytotoxic effects on vero cells [15]. The chromosomal *subAB*_*2*_ variant has been recently shown to be harbored on a genomic island. This 8058 bp Subtilase-Encoding PAI (SE-PAI), is positioned between the tRNA gene *pheV* and the *yjhs* gene, putatively encoding an 9-O-Acetyl N-acetylneuraminic acid esterase in *E. coli* strain ED32. The SE-PAI contains an integrase gene, a *shiA* gene (homologous to the *shiA* gene of the *Shigella flexneri* pathogenicity island SHI-2), a sulphatase, the toxigenic invasion locus A (*tia*) and the *subAB* operon [16,17].

Several authors described the presence of *subAB* mainly in *eae*-negative STEC strains of non-O157 serogroups such as O77, O79, O105 [7], serotype O128:H2 from sheep [18], and a number of other STEC from various origins [16,19,20]. But human cases of infection have also been described [15,16,21,22].

The aim of the current study was to characterize the *subAB* genes and their genetic surrounding in a collection of 18 *subAB*-positive food-borne STEC strains in order to get a more detailed understanding of gene variability, genetic structure, and location.

## Methods

### Bacterial strains and culture conditions

The 18 *subAB* positive STEC strains were isolated between 2008 and 2009 from different food sources in Germany (Table 1). STEC strains were routinely cultured in LB-broth (1% Bacto trypton, 0.5% yeast extract, 1% NaCl, pH 7.4) at 37°C. For solid media, 1.5% Bacto agar was added.

**Table 1:** **Strain characteristics and results of the genetic analysis of different *subAB*-loci and related virulence genes of food-associated STEC**

**Strain**^**a**^	**Serotype**^**a**^	**Source**^**a**^	***Stx*-type**^**a**^	***tia***	***saa***	***subAB***_***1***_	***subAB***_***2-1***_	***subAB***_***2-2***_
K17	O22:H8	Raw milk	2, 2EC-1586, 2NV-206	-	+	+	-	-
LM5602/08	O22:H8	Lime blossom tea	2v-ha	-	+	+	-	-
CB11588	O102:NM	Pork	2v-hb	-	+	+	-	-
CB11633	O179:H8	Mettwurst	2	-	+	+	-	-
TS20/08	O153:HNT	Minced meat	1, 2	-	+	+	-	-
TS26/08	O179:H8	Minced meat	1, 2	+	+	+	-	-
SF16b	ONT:H11	Minced meat	1, 2	-	+	+	-	-
TS18/08	O113:H21	Minced meat	2	-	+	+	-	-
TS30/08	O113:H21	Minced meat	2	-	+	+	-	-
LM27555	OR:NM	Deer meat	2-O118	+	-	-	+	-
LM14960	O23:NM	Deer meat	2-O118	+	-	-	+	-
LM27558_stx1_	O128:HNT	Deer meat	1, 2-O118	+	-	-	+	-
LM14603/08	O21:H21	Deer meat	2-O118	+	-	-	-	+
LM16092/08	O21:H21	Deer meat	2-O118	+	-	-	-	+
LM27553_stx2_	O110:H31	Deer meat	2-O118	+^b^	-	-	-	+
LM27553_stx1_	O75:H8	Deer meat	1c, 2-O118	+^c^	-	-	+^d^	+
LM27564	O113:NM	Deer meat	2-O118	+	-	-	+	+
LM27558_stx2_	OR:H43	Deer meat	2-O118	+	-	-	+	+

^a^data were taken from Slanec et al. [19].

^b,c,d^PCR products with a size of 1300 bp, 2000 bp, and 4500 bp respectively.

+positive PCR result, - negative PCR result.

### Molecular methods

Purification of the large STEC plasmids was performed according to Kado et al. [23], with minor modifications. Chromosomal DNA was prepared according to standard methods [24]. Concentration and purity of plasmid and chromosomal DNA was measured by UV–vis spectrophotometry using a Nanodrop 2000 device (Thermo Scientific, Germany).

For detection of the *subAB* operon on chromosomal or plasmid DNA, a 1066 bp DNA probe spanning the *subA* and *subB* gene region was generated by PCR with a Roche PCR DIG probe synthesis kit (Roche Applied Science, Germany) using the primer pair subAB-V-for and subAB-V-rev (Table 2). Strain TS30/08 was used as a template for *subAB*_*1*_ and strain LM27558_stx2_ was used for *subAB*_*2*_ with the same primer pair. The specificity of the probes were tested by hybridization of the probes with *subAB* genes cloned in vector pK18 [25] (data not shown). The purified chromosomal and plasmid DNA of *subAB*-positive strains was separated on a 0.8% agarose gel with 130 V at 4°C for 2 h. Subsequently, DNA was transferred on a nitrocellulose membrane by vacuum blotting (VacuGene XL, GE Healthcare, USA) at 60 mbar, then treated with a 1% (v/v) blocking solution (Roche Applied Science, Germany) and hybridized at 73°C for 20 hours. The detection protocol was performed according to the manufacturer’s instruction using sheep anti-digoxigenin-AP Fab fragments (Roche Applied Science, Germany).

**Table 2:** Designations, targets, and positions of primers for PCR analysis and Southern blot hybridization

**Primer**	**Target**	**Sequence (5′ – 3′)**	**GenBank accession number**	**Reference**
saaDF	*saa*	5′-CGT GAT GAA CAG GCT ATT GC-3′	AF399919	[26]
saaDR	*saa*	5′-ATG GAC ATG CCT GTG GCA AC-3′	AF399919	[26]
subAB-V-for	*subAB*	5′-CTT CCC TCA TTG CCT CAC G-3′	AY258503	This study
subAB-V-rev	*subAB*	5′-GGC TGG CCT GTT GTG TAA A-3′.	AY258503	This study
tia_lo	*tia*	5′-TCC ATG CGA AGT TGT TAT CA-3′	U20318	[15]
tia_sense	*tia*	5′-TTC TCT TTT TAC CCT GCT TTT TGC-3′	FJ664545	[15]
subAB-for5	*subAB*_*1*_	5′-CGT ATC TGC GCC ATA TCC TG-3′	AY258503	This study
subAB-rev5	*subAB*_*1*_	5′-CTG TTC CGA GCA GCC ATA TC-3′	AY258503	This study
subAB2-3′tia	*subAB*_*2-1*_	5′-ACT GGC TGT TCT AAC CG-3′	AEZO02000028.1	This study
subA_out	*subA*_*2-2*_	5′-GAA TCA ACA ACA GAT ACG AC-3′	AEZO02000020.1	This study
subA-L	Linker^a^	5′-ATG AAT GAG AGC ATC CCT-3′	AEZO02000020.1	This study
subAB5′OEP	*subAB*_*2-2*_	5′-TAA TGT TTT TGA GAC GGG-3′	AEZO02000020.1	This study
subAB2-3′out	*subAB*_*2-2*_	5′-AGG TCG GCT CAG TGT TC-3′	AEZO02000020.1	This study

^a^intergenic linker between the OEP-locus and *subA*_*2-2.*_

### PCR-screening, sequencing and sequence analysis

Characterization, and sequencing of *subAB* alleles as well as the presence of *saa* or *tia* genes were determined by amplification with the oligonucleotides shown in Table 2. DNA sequence analysis of *subAB* open reading frames was carried out by capillary sequencing using a CEQ™ 8000 Genetic Analysis System (Beckman Coulter, Germany) and the CEQ Dye Terminator cycle sequencing (DTCS) quick start kit (Beckman Coulter, Germany) according to the manufacturer’s recommendation. Final DNA sequences were obtained by sequencing both complementary strands with an at least two-fold coverage. Oligonucleotides for sequencing were created using the Oligo-Explorer ver. 1.1.2 software (http://www.genelink.com) using nucleotide sequences of *E. coli* strains 98NK2 (Acc. no. AY258503), ED32 (Acc. no. JQ994271), and 1.02264 (Acc. no. AEZO02000020.1) from the NCBI database. The same sequences were used as reference sequences for phylogenetic analyses and sequence comparison. The obtained sequences for all *subAB* alleles were submitted to the EBI database and achieved consecutive accession no. from #HG324027 - #HG324047. Editing of raw data and sequence-alignments were carried out using Bioedit, version 7.0.5.3 [27]. Phylogenetic analysis of the different *subA* genes was conducted using Mega 5.1 with an UPGMA algorithm [28].

## Results

### Genomic localization of *subAB* genes

**Figure 1:**
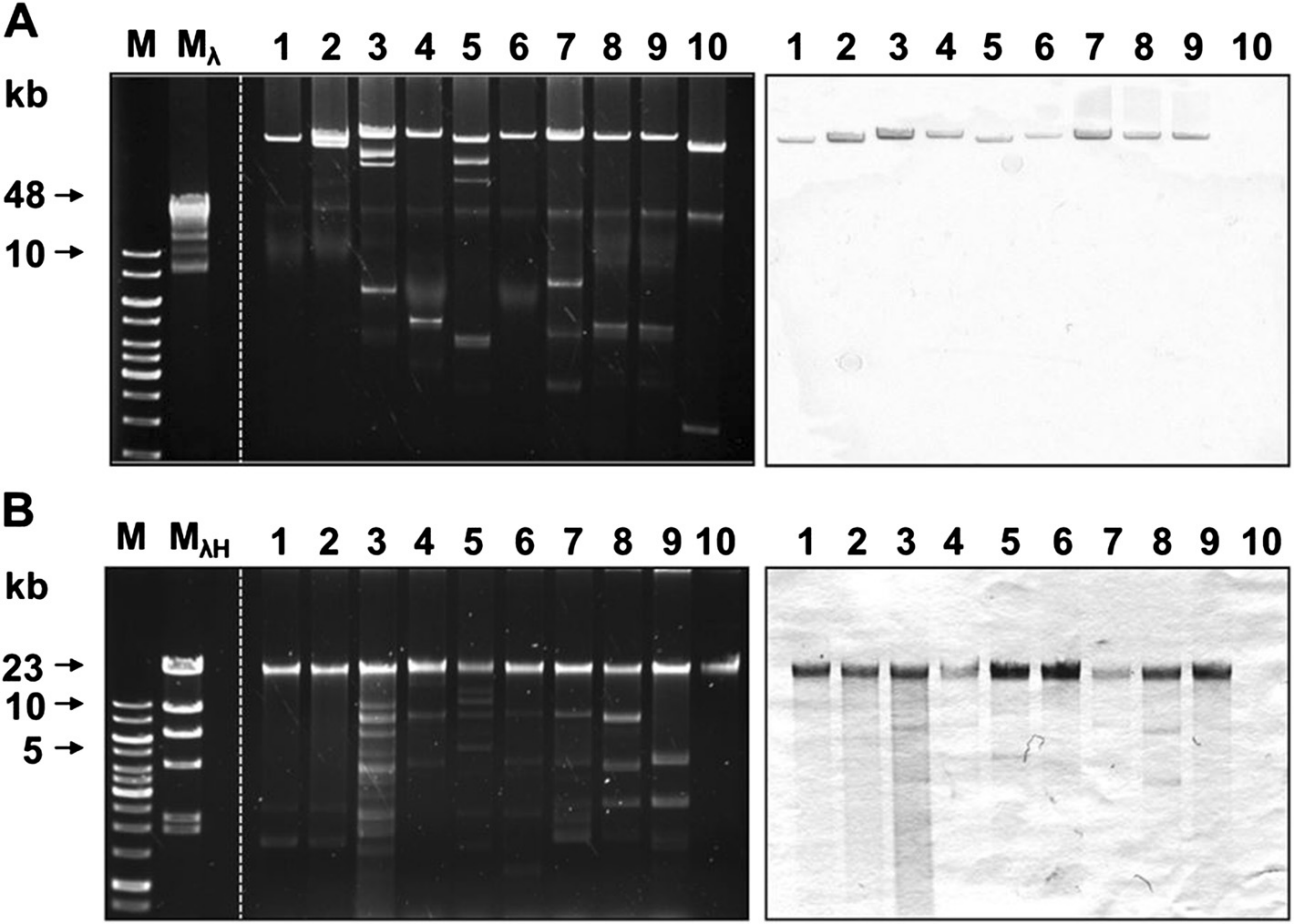
**Agarose gel electrophoresis and Southern blot hybridization of DNA preparations of 18 STEC strains. A)** plasmid preparations (left side) and Southern blot hybridization with a *subAB*_*1*_ specific DNA probe (right side). Gene Ruler 1 kb DNA ladder (M), Lambda-Mix Marker 19 (M_λ_) (both Fermentas), K17 (lane 1), LM25602/08 (2), CB11588 (3), CB11633 (4), TS20/08 (5), TS26/08 (6), SF16b (7) TS18/08 (8), TS30/08 (9), EDL933 (10). **B)** chromosomal DNA (left side) and Southern blot hybridization with a *subAB*_*2*_ specific DNA probe (right side). Gene Ruler 1 kb DNA ladder (M), Lambda DNA/HindIII Marker (M_λH_) (Fermentas), LM14603/08 (1), LM16092/08 (2), LM227553_stx1_ (3), LM227553_stx2_ (4), LM27564 (5), LM27558 (6), LM27555 (7), LM14960 (8), LM27558 (9). EDL933 (10) was used as a negative control for hybridization. Recombinant plasmid pK18 containing *subAB*_*1*_ was used as positive control for hybridization (data not shown).

In order to characterize the *subAB* genes of 18 food-borne STEC from a previous study, which were positive by PCR targeting a fragment of the *subAB* operon [19], they were initially analyzed for the presence and genetic location of their complete ORF. By purification and gel electrophoresis of plasmid DNA of all 18 STEC strains, it could be demonstrated that all strains carried plasmids of various sizes (data not shown). Sixteen strains carried large plasmids with molecular weights larger than that of plasmid pO157 of *E. coli* O157:H7 strain EDL933 (representative plasmid preparations are shown in Figure 1A). Southern blot hybridization with a specific DNA probe directed to *subAB*_*1*_, showed that 9 strains carried *subAB*_*1*_ on a large plasmid (Figure 1A). None of the other strains reacted with the probe (data not shown). Southern blot hybridization of chromosomal DNA preparations (representative DNA preparations are shown in Figure 1B) reacted with a probe directed to *subAB*_2_ and demonstrated chromosomal localization of *subAB*_2_ in the other 9 strains (Figure 1B).

### PCR analysis of *subAB* and adjacent DNA regions

**Figure 2:**
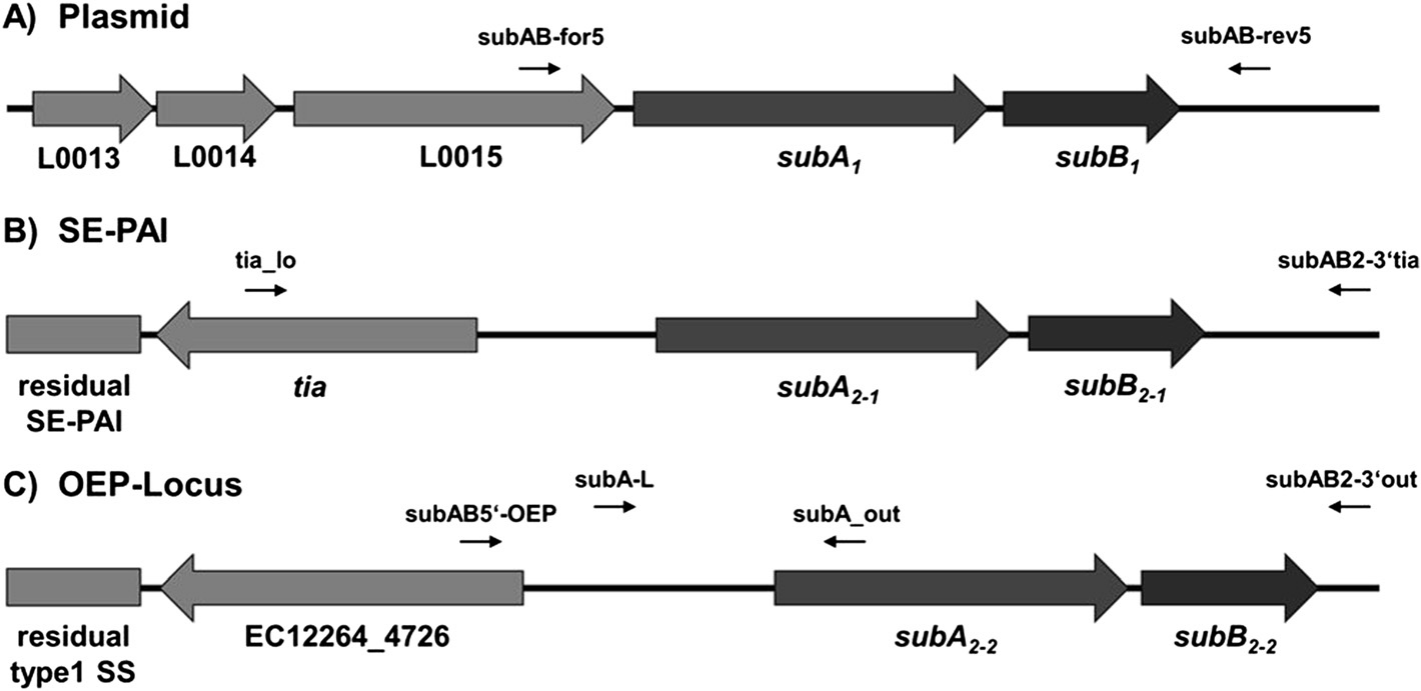
**Schematic illustration of the different genomic loci of *subAB*. A)** plasmid locus of *subAB*_1_ of *E. coli* O113:H21 strain 98NK2 (GenBank Acc. No. AY258503) with three putative genes located upstream of the *subAB* operon and primer binding sites 202 bp upstream and 194 bp downstream of the operon. **B)** genomic locus of *subAB*_*2-1*_ of *E. coli* O78:H^-^ strain ED32 (Acc. No. JQ994271) with the *tia* gene of the SE-PAI located 789 bp upstream of the operon and primer binding sites 1336 bp upstream and 316 bp downstream of the operon. **C)** locus of the new (*subAB*_*2-2*_) operon of *E. coli* O76:H^-^ strain 1.2264 (Acc. No. AEZO02000020.1) with an outer membrane efflux protein as part of a type 1 secretion system located 1496 bp upstream of the *subAB* operon and primer binding sites 1235 bp upstream and 65 bp downstream of the operon. Primers subA-L and subAB2-3′out (Table 1) were used to generate a template for sequencing.

All STEC strains were analyzed by PCR with specific primers directed to the *subAB* operon or flanking regions of the two recently described *subAB* alleles [8,16] (Figure 2). PCR-products were confirmed by DNA-sequencing. For the detection of plasmid-located *subAB*_1_, primer pair subAB-for5/subAB-rev5 (Figure 2A) was used to amplify the complete ORF, including a region 202 bp upstream and 194 bp downstream of *subAB*_1._ The nine strains with plasmid-located *subAB*_*1*_ yielded a PCR product of the expected size of 1821 bp, indicating the presence of the *subAB*_*1*_ variant and complete ORFs in these strains (data not shown). Moreover, *saa* was present in these strains indicating a similar genetic arrangement as previously described [8].

Since it has been reported that the chromosomal *subAB*_*2*_ variant of STEC strain ED32 was linked to the *tia* gene in the chromosomal island SE-PAI [16], corresponding primers were used to test the hypothesis whether the remaining 9 strains contained this particular variant (for a scheme see Figure 2B). In initial experiments PCR with primers tia_lo and tia_sense (Table 2) was positive in all nine strains and proved the presence of the *tia* gene. However, PCR products of strains LM27553_stx1_ and LM27553_stx2_ were larger than expected, indicating insertion of foreign DNA into or closely to the *tia* gene [15] (Table 1).

**Figure 3:**
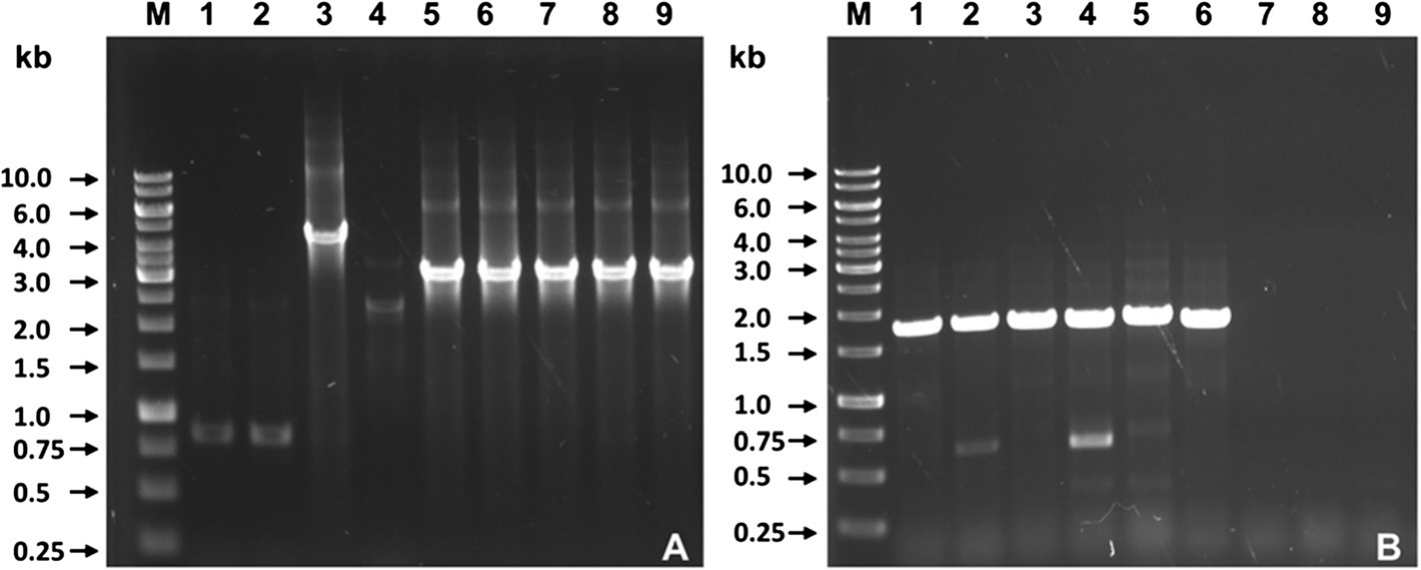
**Agarose gel electrophoresis of PCR products of *subAB***_***2***_**alleles with primers tia_lo/subAB2-3′tia targeting the SE-PAI (A), and subAB5′-OEP/subA_out targeting the OEP-locus locus (B).** Gene Ruler 1 kb DNA ladder (M), (Fermentas) LM14603/08 (1), LM16092/08 (2), LM27553_stx1_ (3), LM27553_stx2_ (4), LM27564 (5), LM27558_stx2_ (6), LM27555 (7), LM14960 (8), LM27558_stx1_ (9), with identical order of strains on both agarose gels. Strain LM27564 was used as positive control.

Following this, the structure of the *subAB*_2_ operon and adjacent DNA was analyzed using the primer pair tia_lo/ SubAB2-3′tia targeting the region of the *tia* gene, an intergenic region (linker), *subAB*_2_, as well as 316 bp of the downstream region (Figure 2B). This should reveal a PCR product of 3174 bp. In these PCRs, 6 STEC strains were positive (see Figure 3A, lanes 3, 5–9), indicating the presence of *subAB*_*2*_ linked to the *tia* gene (Table 1). However, one of these PCRs with strain LM27553_stx1_ as a template, revealed a PCR product of approximately 4500 bp (Figure 3A, lane 3). Since the open reading frames of *subA*_*2-1*_ and *subB*_*2-1*_ in this strain were of the correct size, insertion of foreign DNA between *subA*_*2-1*_ and *tia* is assumed. PCR of STEC strains LM14603/08, LM16092/08 and LM27553_stx2_ with the same primers was negative (Figure 3A, lanes 1, 2, and 4), and therefore direct association of *subAB*_*2*_ with the *tia* gene could not be demonstrated. Weak bands in Figure 3A, lanes 1, 2, and 4 reflect unspecific amplification products.

Due to these negative results, the *subAB*_2_ reference sequence of STEC strain ED32 (GenBank Acc. No. JQ994271) was searched with BLAST against the NCBI nucleotide database to evaluate the possibility of further *subAB* gene loci in these strains. Interestingly, a further *subAB* operon with different flanking regions was detected in *Escherichia coli* strain 1.2264 in contig 3905 (Acc. No. AEZO02000020.1) and in *Escherichia coli* strain 9.0111 in contig 1125855384441 (Acc. No. AEZZ02000028.1), which in addition carry the SE-PAI described by Michelacci et al. [16]. The new gene locus carries genes hypothetically encoding parts of a type 1 secretion system (T1SS), and an outer membrane efflux protein (OEP), which are located upstream of *subAB*_*2*_ and are linked to the latter by a 1496 bp sequence (for a scheme see Figure 2C). Downstream of *subAB*_*2*_*,* the *nanR* gene hypothetically encoding the transcriptional regulator of the *nan*-operon was present in a 1400 bp distance in strain *E. coli* 1.2264 and 3842 bp in *E. coli* 9.011 where additional putative transposases are inserted (data not shown). In the following, this new gene region is termed OEP-locus.

To test the hypothesis whether in the STEC strains investigated here also two copies of *subAB*_*2*_ are present, oligonucleotides SubAB5′-OEP and SubA_out were designed (Figure 2C) and used for PCR amplification of all *subAB*_*2*-_positive strains. Six strains were positive with these primers (Figure 3B, lanes 1–6), including the strains LM14603/08, LM16092/08 and LM27553_stx2_, which were negative for the SE-PAI (Figure 3A, lanes 1,2, and 4). Moreover, this demonstrated that STEC strains LM27553_stx1_, LM27564 and LM27558_stx2_ contained both chromosomal *subAB*_*2*_ loci (Table 1).

### Sequencing of *subAB* open reading frames

In order to further prove that the *subAB* operons contained complete ORFs, we determined the nucleotide sequence of the entire *subAB* open reading frames of the PCR products derived from the three different gene loci. Results of the DNA sequencing complied with the PCR data (see above), and confirmed the presence of three loci encoding different alleles of *subAB*. The different alleles of the chromosomal loci were designated *subAB*_*2-1*_ for the one located in the SE-PAI and *subAB*_*2-2*_ for the new variant located in the OEP-locus. The sequence of the nine *subAB*_*1*_ operons was identical and comprised 1486 bp from the start codon of *subA*_*1*_ to the last base of the stop codon of *subB*_*1*_. Sequences were 99.8% identical to the corresponding *subAB* operon sequence of strain 98NK2 published by Paton et al. [8].

In all 12 chromosomal DNA sequences the A-subunit genes had the same length as the *subA*_*1*_ genes described above and that from reference strain 98NK2. All but one *subB*_*2*_ genes had the same length as the reference sequence of ED32 but were one triplet shorter at the 3′-end of the gene, than *subB*_*1*_. This resulted in the lack of the N-terminal amino acid serine in the putative SubB2-subunits.

Moreover, the *subB*_*2-2*_ sequence of strain LM27553_stx1_ contained an insertion of a single thymine; generating a stretch of 5 T’s at position 1298–1302, which was not present in the *subB*_*2*_ alleles of the other strains. This resulted in a frame shift in the B-subunit gene, and thereby to a stop codon at position 253 of the ORF. This putatively results in a truncated protein of 84 amino acids instead of 140 amino acids as for the full length SubB2 subunits.

**Figure 4:**
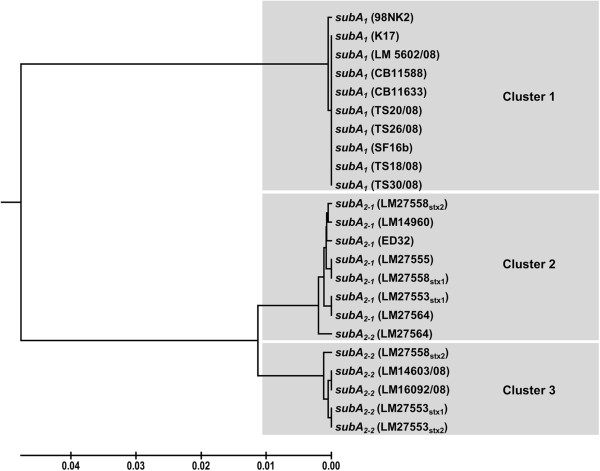
**Sequence analysis and phylogenetic distribution of *subA *alleles from different genomic loci.** Phylogenetic analyses were performed after sequencing and sequence analysis by the software Mega 5.1 using the UPGMA algorithm [28]. Results of the phylogenetic analysis demonstrate clustering of all *subA* genes according to their genomic loci with exception of *subA*_*2-2*_ of strain LM27564 which is located in the region between *subA*_*2-1*_ and *subA*_*2-2*_*.*

Phylogenetic analysis of all 21 A-subunit genes clearly demonstrated three clusters (Figure 4). Cluster 1 comprises the very homogeneous *subA*_*1*_ genes, cluster 2 the *subA*_*2-1*_ genes, including the reference sequence of ED32, and cluster 3 the *subA*_*2-2*_ genes located in the OEP-locus. In cluster 2 there is a single *subA*_*2-2*_ allele located on the OEP-locus (Figure 4).

Comparing the whole *subAB* sequences of 1483 bp (sequences were cut to the same length), the *subAB*_*2-1*_ sequences of cluster 2, including *subAB*_*2-2*_ of strain LM27564 were 99.5% identical to each other. The sequence identities of *subAB*_*2-1*_ to the reference strain ED32 were in a range of 99.2-99.5% for the other *subAB*_*2-1*_ alleles.

The *subAB*_*2-2*_ sequences of the OEP-locus without strain LM27564 (see above) were 99.9% identical to each other and showed sequence homologies of about 91.0% to *subAB*_1_. Moreover, *subAB*_*2-2*_ is 98.4% identical to *subAB*_2-1_ and 99.9% to the reference sequence of the OEP-locus of *E. coli* 1.2264 (Acc. No. AEZO02000020.1).

The *subAB*_*2-2*_ genes of the OEP-locus of strain LM27564 showed 99.1% sequence identity to *subAB*_2-1_ of strain ED32 and only 89.9% with *subAB*_1_ of strain 98NK2 and 97.9% to the OEP-locus of *E. coli* strain 1.2264. The results of these sequence comparisons show that the sequences of the three alleles are conserved but heterogeneity is present between the loci.

## Discussion

The results of this study have shown that those 18 food-borne STEC, which have previously been demonstrated to be *subAB*-positive by PCR [19] carry complete *subAB* open reading frames. Besides the plasmid-locus, as originally described by Paton et al. [8], and the SE-PAI described by Michelacci et al. [16], a new chromosomal region, the OEP-locus, was present in six strains analyzed here and demonstrated to harbor *subAB*_*2-2*_ operons.

It could be shown that all strains contained at least intact open reading frames for one *subAB* operon, and the codons specifying the amino acids constituting the catalytic triad were present in all cases (data not shown). From the sequence data obtained in our study, it can be concluded that all strains are able to produce functional SubAB subtilase cytotoxins.

The STEC strains analyzed in our study with subtilase-encoding plasmids did not carry chromosomal *subAB* genes and vice versa. Up to now we do not know whether this is a basic principle or whether this is only observed in our small strain collection. However, we cannot rule out that chromosomal-encoded and plasmid-encoded *subAB* genes exclude each other or that recombination between plasmids and the chromosome in *subAB*-carrying strains is low. Phylogenetic analyses of the *subA* genes clearly differentiated three clusters, the plasmid-located being the most homogeneous one. The chromosomal clusters showed more genetic diversity, indicating a different phylogenetic history (Figure 4). These phylogenetic differences could reflect a different pathogenic potential and toxicity of *subAB*-positive strains for humans as it was shown for the different Shiga toxin variants [29,30]. Therefore, it could be important to analyze the enzymatic and toxic activity of the variants in different cell culture and animal models. Moreover, it could be important to analyze which *subAB* variants are associated with serious diseases and whether further variants exist, which currently have not been described.

In a former study, it could be shown that the 18 strains used here carried gene fragments of the subtilase cytotoxin [19]. These strains were isolated from different food-sources and showed a high serotype heterogeneity demonstrating the wide spread of *subAB* in *stx*-positive *E. coli*. Genetic analysis of these strains demonstrated that the chromosomal encoded *subAB*_*2*_-positive strains were all associated with deer meat, whereas the plasmid encoded *subAB*_*1*_ could be found in strains from different sources. This association of the chromosomal encoded *subAB*_*2*_ variant with deer was also described in other studies [16,18,31] and suggests the possibility of small ruminants as reservoir for *subAB*_*2*_ positive STEC.

## Conclusions

The results of our analysis have confirmed that *subAB* should be further considered as a marker for virulence, especially in food-borne STEC strains. The occurrence of more than one *subAB* allele in particular strains is interesting and raises the question whether multiple gene acquisitions may bear a selective advantage for those strains. The fact that subtilase cytotoxin-producing *Escherichia coli* have not been frequently involved in outbreaks of human disease could be a hint for a function in other hosts such as small ruminants. Increased detection of *subAB* in such animals supports this assumption. However, cell culture and animal experiments have shown profound toxic effects on primary human epithelial cells [32]. Therefore, future studies are necessary to investigate the function and expression of the different *subAB* alleles in more detail.

## Competing interests

The authors declare that they have no competing interests.

## Authors’ contributions

JF carried out the practical experimental laboratory work, participated in the sequence alignment and drafted the manuscript. HSt participated in the design of the study and helped to draft the manuscript. EH participated in the sequence analysis and alignment. HS conceived of the study, participated in its design and coordination, helped to draft the manuscript, and gave final approval of the version to be published. All authors read and approved the final manuscript.
