# Correction: Molecular analysis of subtilase cytotoxin genes of food-borne Shiga toxin-producing *Escherichia coli* reveals a new allelic *subAB* variant

**DOI:** 10.1186/1471-2180-14-32

**Published:** 2014-02-10

**Authors:** Joschua Funk, Helen Stoeber, Elisabeth Hauser, Herbert Schmidt

**Affiliations:** 1 Department of Food Microbiology, Institute of Food Science and Biotechnology, University of Hohenheim, Garbenstraße 28, D-70599 Stuttgart, Germany

## Correction

After publication of this work [1], we noticed that the sequences of primers subA-L and subAB5’-OEP in Table two (Table 1 here) are not correct. We therefore provide the correct primer sequences in a new Table two (Table 1 here).

**Table 1:** Designations, targets, and positions of primers for PCR analysis and Southern blot hybridization

**Primer**	**Target**	**Sequence (5′ – 3′)**	**GenBank accession number**	**Reference**
saaDF	*saa*	5′-CGT GAT GAA CAG GCT ATT GC-3′	AF399919	[26]
saaDR	*saa*	5′-ATG GAC ATG CCT GTG GCA AC-3′	AF399919	[26]
subAB-V-for	*subAB*	5′-CTT CCC TCA TTG CCT CAC G-3′	AY258503	This study
subAB-V-rev	*subAB*	5′-GGC TGG CCT GTT GTG TAA A-3′	AY258503	This study
tia_lo	*tia*	5′-TCC ATG CGA AGT TGT TAT CA-3′	U20318	[15]
tia_sense	*tia*	5′-TTC TCT TTT TAC CCT GCT TTT TGC-3′	FJ664545	[15]
subAB-for5	*subAB*_*1*_	5′-CGT ATC TGC GCC ATA TCC TG-3′	AY258503	This study
subAB-rev5	*subAB*_*1*_	5′-CTG TTC CGA GCA GCC ATA TC-3′	AY258503	This study
subAB3′tia	*subAB*_*2-1*_	5′-ACT GGC TGT TCT AAC CG-3′	AEZO02000028.1	This study
subA_out	*subA*_*2-2*_	5′-GAA TCA ACA ACA GAT ACG AC-3′	AEZO02000020.1	This study
subA-L	Linker^a^	5′-GGG AGG ATT AAC CAT CG-3′	AEZO02000020.1	This study
subAB5′OEP	*subAB*_*2-2*_	5′-ATG AAT GAG AGC ATC CCT-3′	AEZO02000020.1	This study
subAB2-3′out	*subAB*_*2-2*_	5′-AGG TCG GCT CAG TGT TC-3′	AEZO02000020.1	This study

^a^Intergenic linker between the OEP-locus and *subA*_*2-2*_.
